# Endodermal pouch-expressed *dmrt2b* is important for pharyngeal cartilage formation

**DOI:** 10.1242/bio.035444

**Published:** 2018-10-19

**Authors:** Linwei Li, Aihua Mao, Peng Wang, Guozhu Ning, Yu Cao, Qiang Wang

**Affiliations:** 1 State Key Laboratory of Membrane Biology, CAS Center for Excellence in Molecular Cell Science, Institute of Zoology, University of Chinese Academy of Sciences, Chinese Academy of Sciences, Beijing 100101, China; 2 Institute for Stem Cell and Regeneration, Chinese Academy of Sciences, Beijing 100101, China

**Keywords:** *dmrt2b*, Endodermal pouch, Craniofacial cartilage, *cxcl12b*, *crossveinless 2*

## Abstract

Pharyngeal pouches, a series of outpocketings derived from the foregut endoderm, are essential for craniofacial skeleton formation. However, the molecular mechanisms underlying endodermal pouch-regulated head cartilage development are not fully understood. In this study, we find that zebrafish *dmrt2b*, a gene encoding Doublesex- and Mab-3-related transcription factor, is specifically expressed in endodermal pouches and required for normal pharyngeal cartilage development. Loss of *dmrt2b* doesn't affect cranial neural crest (CNC) specification and migration, but leads to prechondrogenic condensation defects by reducing *cxcl12b* expression after CNC cell movement into the pharyngeal arches. Moreover, *dmrt2b* inactivation results in reduced proliferation and impaired differentiation of CNC cells. We also show that *dmrt2b* suppresses *crossveinless 2* expression in endodermal pouches to maintain BMP/Smad signaling in the arches, thereby facilitating CNC cell proliferation and chondrogenic differentiation. This work provides insight into how transcription factors expressed in endodermal pouches regulate pharyngeal skeleton development through tissue–tissue interactions.

## Abstract (precis)

**Summary:** Transcription factor *dmrt2b* facilitates *cxcl12b* expression in endodermal pouches to accelerate cranial neural crest cell condensation, but suppresses *crossveinless 2* expression for supporting cell growth and chondrogenic differentiation.

## INTRODUCTION

Craniofacial malformations, which occur due to developmental issues of the head, face and neck, account for approximately one-third of congenital birth defects (DeLuke, 2014). Owing to the ceaseless efforts of scientists, more than 700 distinct craniofacial syndromes have been described (Johnson and Wilkie, 2011; Lisa and Elden, 2014; Schutte and Murray, 1999). The neurocranium is derived from both the cranial neural crest (CNC) and mesoderm, while the pharyngeal skeleton, including the jaw and branchial arches, is solely derived from CNC cells (Yelick and Schilling, 2002). CNC cells emerge from the dorsal and lateral regions of the neural ectoderm when the epidermal ectoderm interacts with the neural plate to induce formation of the neural plate border (Donoghue et al., 2008). Subsequently, bilateral CNC cells migrate medially with the developing head and then from the midbrain and hindbrain as three streams of collective cell populations (mandibular, hyoid and branchial) into the pharyngeal arches to form the pharyngeal cartilages (Couly et al., 1993; Köntges and Lumsden, 1996; Lumsden et al., 1991; Schilling and Kimmel, 1994).

The craniofacial complex comprises cells from all three germ layer origins: ectodermal, endodermal and mesodermal, and craniofacial morphogenesis requires continuous and reciprocal tissue–tissue interactions (Chai and Maxson, 2006). In particular, in the pharyngeal arches, the CNC with mesoderm core is separated with endodermal pouch inner and covered with epidermal ectoderm outer (Noden, 1988). Endodermal pouches are a series of outpocketings developed in an anterior–posterior wave from the pharyngeal endoderm. Interestingly, although CNC cells are not required for the formation of endodermal pouches (Veitch et al., 1999), these pouches have signaling functions important for the development of the pharyngeal skeleton. Zebrafish mutants, such as *casanova/sox32* and *faust/gata5*, which lack early endoderm and the pharyngeal pouches, exhibit severe defects in craniofacial chondrogenesis, suggesting the endodermal requirements in pharyngeal skeletal development (Dickmeis et al., 2001; Kikuchi et al., 2001; Reiter et al., 1999). Endodermal pouch-derived FGF and BMP ligands have been shown to be required for the survival, proliferation and differentiation of postmigratory CNC cells that give rise to branchial cartilages (Crump et al., 2004; David et al., 2002; Holzschuh et al., 2005; Ning et al., 2013; Nissen et al., 2003). In addition, the T-box transcription factor Tbx1 is a key molecule in the regulation of tissue–tissue interactions (Choe and Crump, 2014; Huh and Ornitz, 2010; Kopinke et al., 2006; Okada et al., 2016; Okubo et al., 2011; Piotrowski et al., 2003). *tbx1* is expressed in the endodermal pouches as well as the mesodermal core of the pharyngeal arches (Piotrowski et al., 2003). In *vgo/tbx1* mutants, the pharyngeal pouches are largely absent, and the pharyngeal cartilages are misshapen and fused together (Piotrowski and Nüsslein-Volhard, 2000). Yet while mesodermal Tbx1 has been shown to function in shaping the lower jaw (Aggarwal et al., 2010), transplantation of wild-type endoderm into *vgo/tbx1* mutants partial rescue the formation of pharyngeal cartilages, indicating that *tbx1* acts non-autonomously in the endoderm (Piotrowski et al., 2003). The identification of new chondrogenic regulators with endodermal expression will promote our understanding of the tissue–tissue interactions during craniofacial skeleton development.

The *doublesex/mab-3 related* (*Dmrt*) gene family consists of transcription factors with a DSX/MAB-3 (DM) domain, which is a zinc finger-like DNA binding motif first identified in the sexual regulatory proteins Doublesex (DSX) and MAB-3 (Erdman and Burtis, 1993; Raymond et al., 1998). There are multiple *dmrt* paralogs in the animal kingdom, but most Dmrt proteins display little similarity with the exception of their DM domain (Volff et al., 2003). These *dmrt* genes have different spatial-temporal expression, suggesting they could have additional functions besides sex determination (Hodgkin, 2002; Hong et al., 2007; Lints and Emmons, 2002; Volff et al., 2003). There are five *dmrt* genes, designated *drmt1*, *dmrt2a*, *dmrt2b*, *dmrt3* and *dmrt5*, in zebrafish. The *dmrt2a* and *dmrt2b* genes originated from the second round of genome duplication, and *dmrt2a* is the homolog of *Dmrt2* that is involved in somitogenesis in vertebrates (Lu et al., 2017; Matsui et al., 2012; Meng et al., 1999; Sato et al., 2010; Saúde et al., 2005; Seo et al., 2006). Interestingly, *dmrt2b* is expressed in the pharyngeal region (Johnsen and Andersen, 2012; Zhou et al., 2008), indicating its potential role in the development of the branchial skeleton.

In this study, we find that zebrafish *dmrt2b* is uniquely expressed in endodermal pouches and reveal a function for this gene in regulating endodermal expression of *cxcl12b* and *crossveinless 2* (*cv2*) to promote pharyngeal CNC cell condensation, proliferation and differentiation. Therefore, this study uncovers a molecular mechanism for regulation of craniofacial cartilage development through tissue–tissue interactions mediated by endodermal pouch-expressed transcription factor Dmrt2b.

## RESULTS

### Zebrafish *dmrt2b* is specifically expressed in pharyngeal pouches

**Fig. 1.**
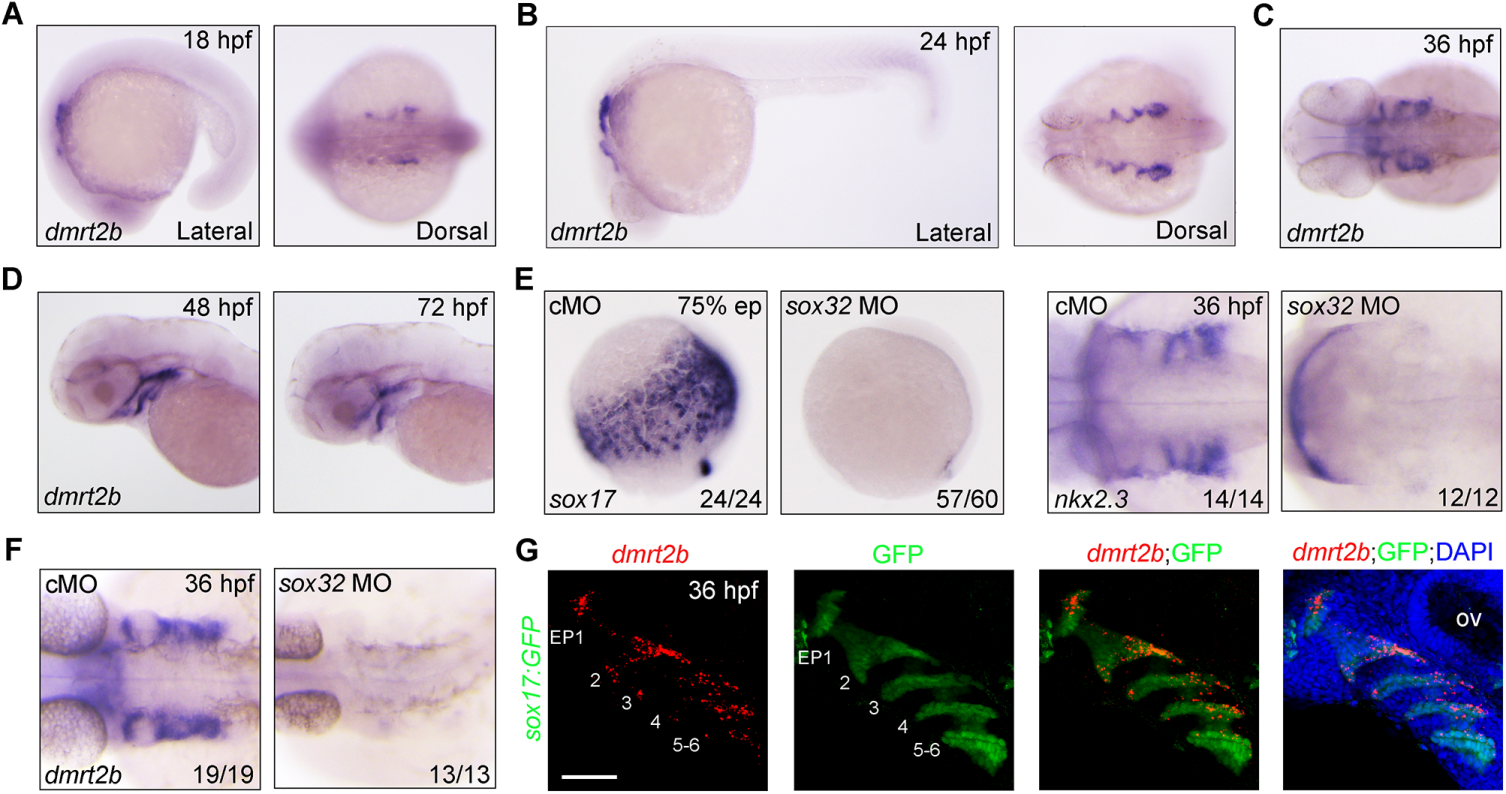
**Expression of *dmrt2b* in the developing endodermal pouches.** (A–D) Analysis of *dmrt2b* expression at different stages. (E,F) Endodermal cells were absent from *sox32* morphants. Expression of endodermal marker *sox17* (E), endodermal pouch marker *nkx2.3* (E) and *dmrt2b* (F) were examined by *in situ* hybridizations at the indicated stages in wild-type embryos injected with 8 ng control MO (cMO) or *sox32* MO. (G) Expression of *dmrt2b* in endodermal pouches. At 36 hpf, *Tg(sox17:GFP)* transgenic embryos were stained for *dmrt2b* mRNA with Dr-*dmrt2b*-C3 probe (red), and then immunostained with anti-GFP antibody (green). Nuclei were counterstained with DAPI (blue). The six endodermal pouches are labeled in the left two panels. EP, endodermal pouch; ov, otic vesicle. Scale bar: 50 µm.

To evaluate the developmental functions of *dmrt2b*, we first examined its spatiotemporal expression during zebrafish embryonic development using *in situ* hybridizations with an anti-sense probe targeting the cDNA sequence downstream the coding region of DM domain. We found that *dmrt2b* was uniquely expressed in the pharyngeal region as early as 18 hours post-fertilization (hpf), when the first endodermal pouch budded (Fig. 1A). During later stages, *dmrt2b* expression spread to the bilateral side of the head in a thread-like manner (Fig. 1B–D), suggesting that *dmrt2b* is expressed in the endodermal pouches. To examine this, 8 ng *sox32* morpholino (MO) was injected into embryos at the one-cell stage, which resulted in the elimination of the entire endoderm and endoderm-derived pouches as indicated by *sox17* and *nkx2.3* expression (Fig. 1E). As expected, *dmrt2b* expression disappeared from the *sox32* morphants (Fig. 1F). Furthermore, RNAscope *in situ* hybridization combined with immunofluorescence was employed to figure out the exact expression pattern of *dmrt2b* in the *Tg(sox17:GFP)* transgenic fish embryos (Chung and Stainier, 2008). As shown in Fig. 1G, *dmrt2b* transcripts co-localized with GFP-expressing endodermal pouches at 36 hpf. These observations strongly suggest that *dmrt2b* is expressed specifically in the pharyngeal pouches.

### Loss of *dmrt2b* causes malformation of pharyngeal cartilages

**Fig. 2.**
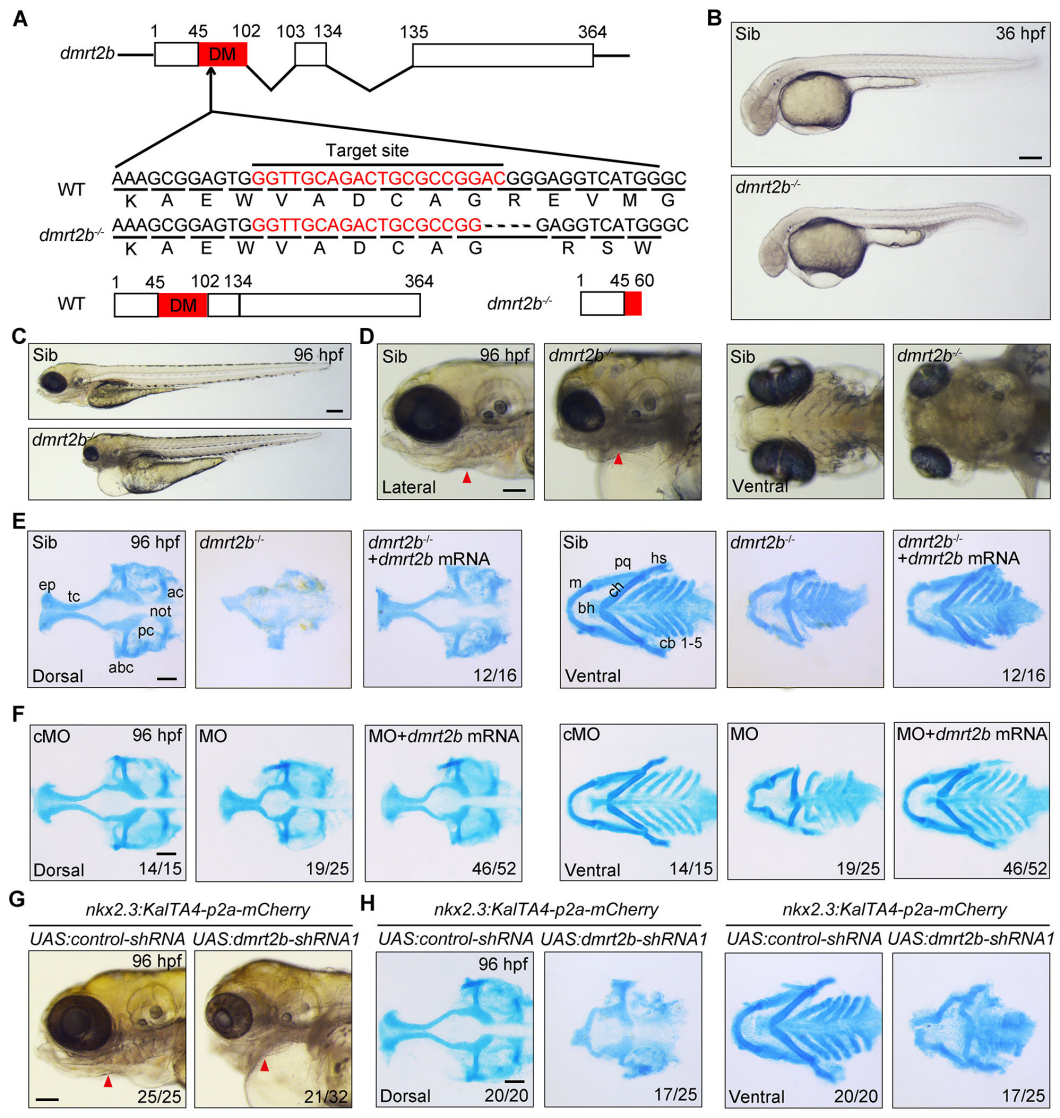
**Depletion of *dmrt2b* impairs cranial cartilage development.** (A) Generation of *dmrt2b* mutant using the CRISPR/Cas9 system. The *dmrt2b* mutant has a four base deletion that results in expression of a truncated protein lacking the DM domain. (B,C) Morphological defects in *dmrt2b* mutants at the indicated stages. (D) Anatomy of the pharyngeal arches and head skeleton in *dmrt2b* mutants. Red arrowheads indicate branchial arches. (E,F) Alcian Blue staining of head cartilages at 96 hpf. Cartilage defects in *dmrt2b* mutants and morphants were abrogated by injection of *dmrt2b* mRNA (F). (G) Anatomy of the pharyngeal arches and head skeleton in embryos injected with indicated shRNA expression plasmids. Red arrowheads indicate branchial arches. (H) Alcian Blue staining of head cartilages in shRNA expression plasmid injected embryos. Scale bar: 100 µm. ac, auditory capsule; not, notochord; pc, parachordal; abc, anterior basicranial commissure; ep, ethmoid plate; tc, trabeculae cranii; m, Meckel's cartilage; bh, basihyal; ch, ceratohyal; pq, palatoquadrate; hs, hyosymplectic; cb, ceratobranchial. Scale bars: 200 µm (B,C), 100 µm (D–H).

To investigate whether *dmrt2b* has functions in pharyngeal pouch formation and craniofacial cartilage development, we mutated the *dmrt2b* gene using the CRISPR-Cas9 system. Because the DM domain enables *dmrt2b* to act as a transcription factor, we targeted this domain and obtained one mutant with a four base frameshift deletion in *dmrt2b*, which led to a premature stop codon (Fig. 2A; Fig. S1A). Furthermore, the obvious reduction of *dmrt2b* transcripts in *dmrt2b* homozygous mutants confirmed the loss of function of this gene (Fig. S1B). In comparison to wild-type and heterozygous siblings, *dmrt2b* homozygous mutants had shrunken heads, pericardial edema and smaller jaws (Fig. 2B,C). In addition, the *dmrt2b* mutants had restricted protruding jaws due to poor pharyngeal arch outgrowth (Fig. 2D). Alcian Blue staining revealed severe reduced and dysmorphic neurocranium cartilages and pharyngeal arch-derived chondrogenic elements in these mutants, suggesting chondrogenic differentiation defects (Fig. 2E). Importantly, these cartilages were recovered by injection of 475 pg *dmrt2b* mRNA into *dmrt2b* mutants (Fig. 2E).

To further confirm the role of *dmrt2b* in these pharyngeal cartilage defects, knockdown experiments were performed using antisense MOs. Specifically, 4 ng of *dmrt2b* MO targeting the intron-exon boundary of the first intron and the second exon of the *dmrt2b* gene was injected into one-cell stage embryos. This resulted in the elimination of endogenous mature *dmrt2b* mRNA and the emergence of interfered mRNA products in the morphants (Fig. S2A), indicating the *dmrt2b* MO is specific and effective. Similar to the *dmrt2b* mutants, the knockdown morphants had obvious defects in head cartilage formation, which were abrogated by co-injection of *dmrt2b* mRNA (Fig. 2F; Fig. S2B). Interestingly, the pericardial edema in the morphants was also alleviated by *dmrt2b* mRNA injection, indicating that *dmrt2b* might function in cardiac development (Fig. S2B). miR30-based short hairpin RNAs (shRNAs) from tissue specific promoters displayed very efficient knockdown of gene expression in eucaryotic organisms (Stegmeier et al., 2005; Zeng et al., 2005). To explore tissue-specific roles of *dmrt2b*, we utilized the KalTA4-UAS system to drive the expression of miR30-based shRNAs (*dmrt2b-*shRNA1 and *dmrt2b*-shRNA2) against two different regions of *dmrt2b* transcripts. By using the Tol2 transposon, we generated a *Tg(nkx2.3:KalTA4-p2a-mCherry)* transgenic zebrafish line with a 5.5 kb *nkx2.3* promoter that could specifically drive the expression of KalTA4 activators and red fluorescent proteins in endodermal pouches (Choe et al., 2013), which were indicated by *Tg(sox17:GFP)* embryos at 36 hpf (Fig. S3A). As shown in Fig. S3B, co-injection of 50 pg *UAS:dmrt2b-shRNA1* plasmid with 100 pg Tol2 transposase mRNA into one-cell stage *Tg(nkx2.3:KalTA4-p2a-mCherry)* embryos led to an obvious decrease of *dmrt2b* expression in endodermal pouches compared with control embryos (Fig. S3B). Importantly, the inactivation of *dmrt2b* in pouches resulted in obvious defects in head cartilage formation (Fig. 2G,H). Thus, endodermal pouch-expressed *dmrt2b* is important for craniofacial cartilage development. In addition, tissue specific depletion of *dmrt2b* by injection of *UAS:dmrt2b-shRNA1* plasmid into *Tg(nkx2.3:KalTA4-p2a-mCherry)* embryos resulted in obvious pericardial edema, implying a non-cell autonomous role of pharyngeal endodermal *dmrt2b* during heart development (Fig. 2G). Interestingly, more severe pharyngeal cartilage defects were observed in *dmrt2b* MO or *UAS:dmrt2b-shRNA1* plasmid injected embryos than *dmrt2b* mutants, indicating that a compensatory protective response against the loss of *dmrt2b* may be to some extent activated in the mutants (Rossi et al., 2015; Wei et al., 2017).

### Inactivation of *dmrt2b* results in disorganized pharyngeal arches

To delineate the mechanisms underlying pharyngeal cartilage defects in the absence of functional *dmrt2b*, we examined the expression of several different markers over the course of embryonic development. *In situ* hybridization revealed the CNC specification marker *foxd3* was expressed in a similar manner in control embryos and *dmrt2b* mutants at the five somite stage (Fig. S4A). This demonstrates that *dmrt2b* is not required for the specification of the CNC. In control embryos, *dlx2a* was expressed in the three CNC groups at 18 and 24 hpf. A slight decrease of *dlx2a* expression was observed at 18 hpf in *dmrt2b* mutants, but subsequently recovered at 24 hpf, indicating that *dmrt2b* is not essential for CNC cell migration into the pharyngeal arches (Fig. S4B,C).

**Fig. 3.**
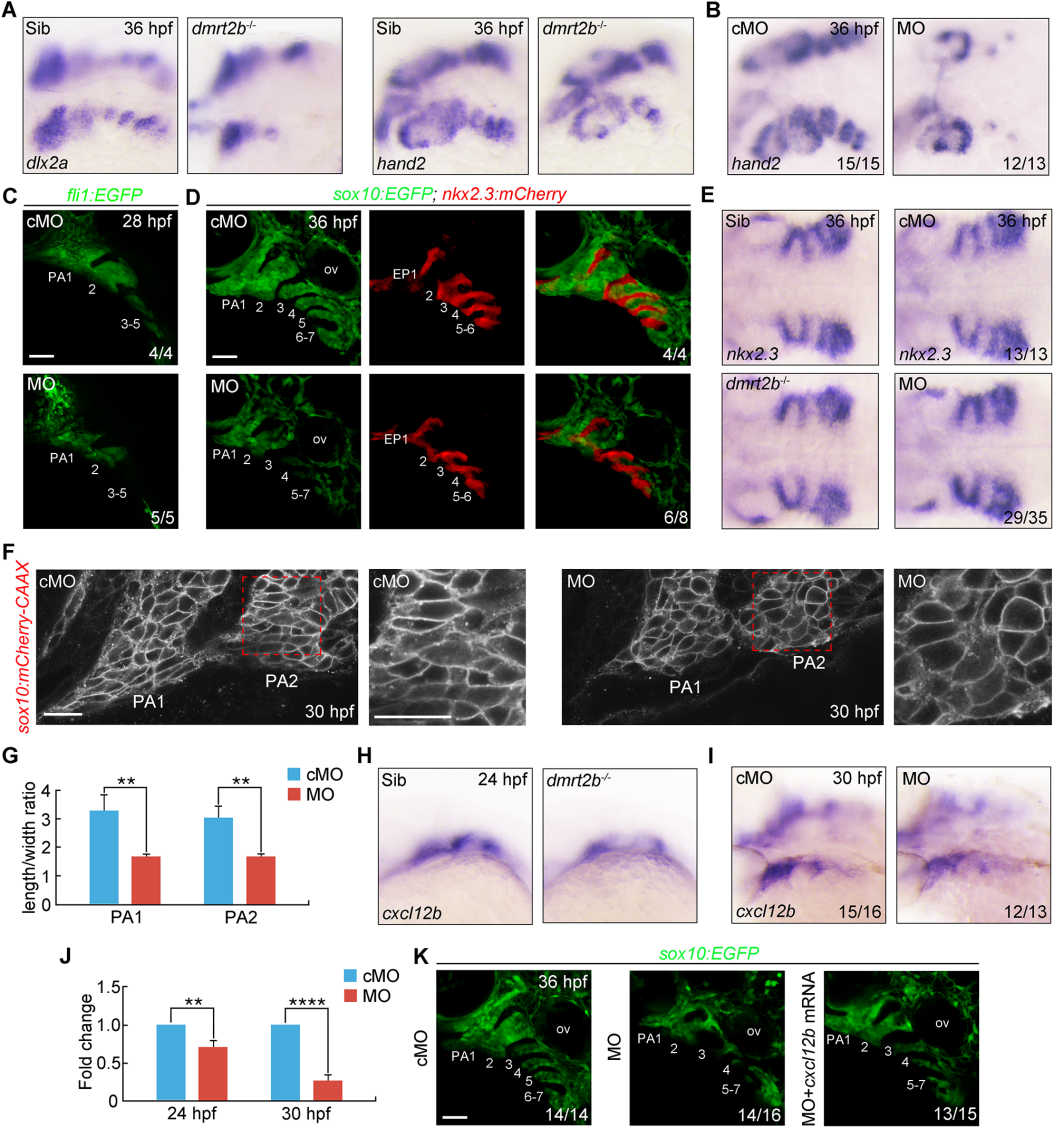
***dmrt2b* functions in CNC cell condensation.** (A,B) Depletion of *dmrt2b* resulted in fewer CNC cells in the pharyngeal arches. *dmrt2b* mutants (A) and morphants (B) were harvested at 36 hpf for *in situ* hybridization with *dlx2a* and *hand2* probes. Lateral views of embryos presented with anterior to the left. (C) Live confocal images of *Tg(fli1:EGFP)* transgenic embryos injected with 4 ng cMO or *dmrt2b* MO at 24 hpf. The pharyngeal arches are numbered. (D) Live confocal images of endodermal pouches and CNC cells in the pharyngeal regions of *Tg(nkx2.3:mCherry; sox10:EGFP)* transgenic embryos at 36 hpf. (E) The expression of endodermal pouch marker *nkx2.3* in *dmrt2b* mutants and morphants. Dorsal views with anterior to the left. (F) Changes in cell shape in the leading edge of the first and second pharyngeal arches in *Tg(sox10:mCherry-CAAX)* embryos injected with 4 ng *dmtr2b* MO. The boxed areas are presented at a higher magnification in the right panels. (G) Quantitation of length/width ratio of CNC cells in the leading edge of the pharyngeal arches. All data are presented as the mean of three independent experiments. Error bars represent s.d. Significance was analyzed using unpaired *t*-tests. **, *P*<0.01. (H,I) The expression of *cxcl12b* in the developing pouches of *dmrt2b* mutants (H) and morphants (I) at the indicated stages. (J) The expression of *cxcl12b* in the head of *dmrt2b* morphants were examined by qRT-PCR at the indicated stages. All data are presented as the mean of three independent experiments. Error bars represent s.d. Significance was analyzed using unpaired *t*-tests. **P<0.01; ****P<0.0001. (K) Live confocal images of CNC cells in the pharyngeal regions of *Tg(sox10:EGFP)* transgenic embryos at 36 hpf. EP, endodermal pouch; ov, otic vesicle; PA, pharyngeal arch. Scale bars: 50 µm (C,D,K), 20 µm (F).

Subsequently, the pharyngeal endoderm migrates laterally to form pouches that interdigitate each of the pharyngeal arches (Crump et al., 2004), then the postmigratory CNC cells in the arches start condensation and proliferation processes and finally differentiate into chondrocytes (Clouthier and Schilling, 2004; Hall and Miyake, 1995; Hall and Miyake, 2000). As *dmrt2b* continues to express in the endodermal pouches after CNC cells reach their destination in the arches, we then examined the arch morphology in *dmrt2b* depleted embryos via the expression of two markers of postmigratory crest, *dlx2a* and *hand2*, at 36 hpf. Compared with control embryos, there were notably fewer CNC cells in *dmrt2b* mutants and morphants (Fig. 3A,B). Similar phenotypes were observed in *dmrt2b* MO-injected *Tg(fli1:GFP)* embryos expressing GFP in CNC derivatives at 28 hpf (Fig. 3C) (Lawson and Weinstein, 2002). Furthermore, *dmrt2b* morphants exhibited loose and disorganized anterior arch structures and the CNC cells failed to aggregate toward certain centers (Fig. 3C). Moreover, injection of 4 ng *dmrt2b* MO into *Tg(nkx2.3:mCherry; sox10:EGFP)* embryos (Carney et al., 2006) resulted in no obvious defects in mCherry-expressing pharyngeal pouches at 36 hpf, and the endodermal pouch marker *nkx2.3* was normally expressed in both *dmrt2b* mutants and morphants (Fig. 3D,E). In contrast, *dmtr2b* depletion gave rise to a similar disorganized arch phenotype and notably fewer GFP-positive CNC cells (Fig. 3D). Moreover, the CNC cells in the pharyngeal region were flattened and elongated, reflecting condensation defects in 4 ng *dmrt2b* MO injected *Tg(sox10:mCherry-CAAX)* embryos, in which cell shape was outlined through plasma membrane-bound mCherry (Fig. 3F,G). Taken together, these findings reveal that *dmrt2b* is dispensable for endodermal pouch morphogenesis, but plays an important role in controlling prechondrogenic condensation in the pharyngeal arches.

Because Dmrt2b is a transcription factor specifically expressed in the pharyngeal endoderm, the non-autonomous activity of *dmrt2b* should be mediated by some secretory molecules that are derived from endodermal pouches and able to regulate pharyngeal arch development. It has been shown that chemokine Cxcl12b signaling from the endodermal pouches is required for the proper condensation of Cxcr4a expressing CNC cells in pharyngeal arches (Boer et al., 2015; Olesnicky Killian et al., 2009). Therefore, we speculate that *dmrt2b* might regulate the expression of *cxcl12b* in pouches to control the prechondrogenic condensation. In support of this hypothesis, after CNC cells migrating from the brain into the pharyngeal arches, both genetic depletion and knockdown of *dmrt2b* resulted in a significant reduction of *cxcl12b* expression in the pharyngeal region (Fig. 3H–J). In addition, the condensation defects of GFP-positive CNC cells were partially rescued by co-injection of 20 pg *cxcl12b* mRNA into *dmrt2b* morphants (Fig. 3K). Therefore, we conclude that *dmrt2b* is a positive regulator of *cxcl12b* in endodermal pouches and thereby drives CNC cell compaction in the pharyngeal arches.

### The proliferation and chondrogenic differentiation of CNC cells require *dmrt2b*

After migrating into the pharyngeal arches, CNC cells proliferate and differentiate into chondrocytes (Hall and Miyake, 2000; Ning et al., 2013). To dynamically observe pharyngeal cartilage defects induced by *dmrt2b* inactivation, *in vivo* time-lapse imaging of CNC cells was performed on *Tg(fli1:EGFP)* embryos. In the control MO (cMO)-injected embryos, GFP-positive CNC cells aggregated as prechondrogenic condensations at 48 hpf, differentiated into chondrocytes at 60 hpf, and organized into chondrocyte stacks from 72 to 84 hpf (Fig. S5). However, in addition to the condensation defects in the first and second pharyngeal arches in *dmrt2b* morphants, there were significantly fewer CNC cells at 48 hpf (Fig. S5). Moreover, only a few CNC cells were observed in pharyngeal arches 3–7 (Fig. S5), consistent with the previously noted cell number reduction in the *dmrt2b* morphants (Fig. 3C,D). These posterior segments emerged at 60 hpf, but were much smaller (Fig. S5). At 72 and 84 hpf, the palatoquadrate and ceratohyal cartilages were shorter and the chondrocytes failed to stack (Fig. S5). These observations are consistent with the Alcian Blue staining results (Fig. 2E).

**Fig. 4.**
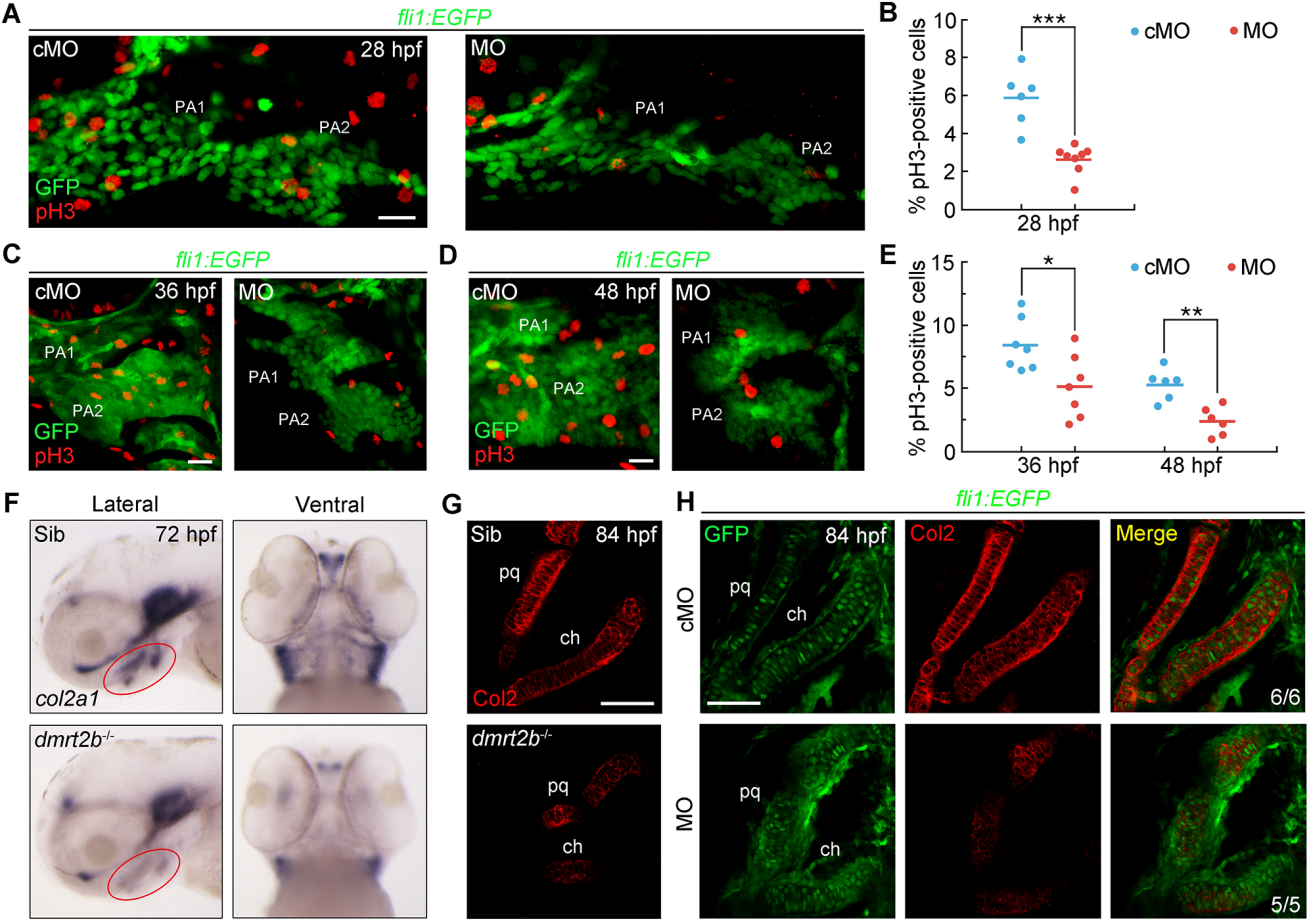
***dmrt2b* facilitates CNC cell proliferation and differentiation.** (A,C,D) Representative confocal sections of pH3-positive cells in the first and second pharyngeal arches at the indicated stages. (B,E) Percentage of pH3-positive cells among GFP-positive CNC cells. *n*≥6 embryos for each condition. Significance was analyzed using unpaired *t*-tests. *P<0.05; **P<0.01; ***P<0.001. (F) Expression levels of *col2a1* mRNA at 72 hpf. Red circles indicate the pharyngeal region. The left panels are lateral views with anterior to the left and the right panels are ventral views with the anterior to the top. (G,H) Immunostaining of Col2 protein in pharyngeal cartilages at 84 hpf. *dmrt2b* mutants (G) and *Tg(fli1:EGFP)* embryos injected with 4 ng *dmrt2b* MO (H) were stained with the indicated fluorescent antibodies. PA, pharyngeal arch; pq, palatoquadrate; ch, ceratohyal. Scale bars: 20 µm.

The reduction in the size of the pre-chondrogenic segments in the *dmrt2b* morphants raises the possibility that cell proliferation and/or survival are inhibited. Therefore, *Tg(fli1:EGFP)* embryos were immunostained for phosphorylated histone 3 (pH3) to assess CNC cell proliferation at 28, 36 and 48 hpf. We found notably fewer mitotic pH3-positive CNC cells in *dmrt2b* morphants than control embryos (Fig. 4A­–E). Conversely, TUNEL revealed a lack of apoptotic cells in the pharyngeal region in *dmrt2b* mutants and morphants (Fig. S6A,B). These findings support that loss of *dmrt2b* impairs CNC cell proliferation, which contributes to the reduction in chondrocyte number within the pharyngeal arches.

Due to the disordered arrangement of the chondrocytes in the absence of *dmrt2b*, we inferred that *dmrt2b* also has an essential role in CNC cell differentiation into chondrocytes. To test this, we assessed the expression of *col2a1*, the gene encoding type II collagen, the primary cartilage matrix protein produced by mature chondrocytes (Vandenberg et al., 1991; Yan et al., 2002). There were dramatically fewer *col2a1* transcripts in the pharyngeal region of *dmrt2b* mutants (Fig. 4F). Meanwhile, Col2 protein levels were also reduced and displayed discontinuous distribution in *dmrt2b-*depleted embryos (Fig. 4G,H). Therefore, loss of *dmrt2b* disrupts chondrogenic differentiation of CNC cells.

### *dmrt2b* maintains BMP signaling through inhibiting *crossveinless 2* to facilitate CNC cell proliferation and chondrogenic differentiation

**Fig. 5.**
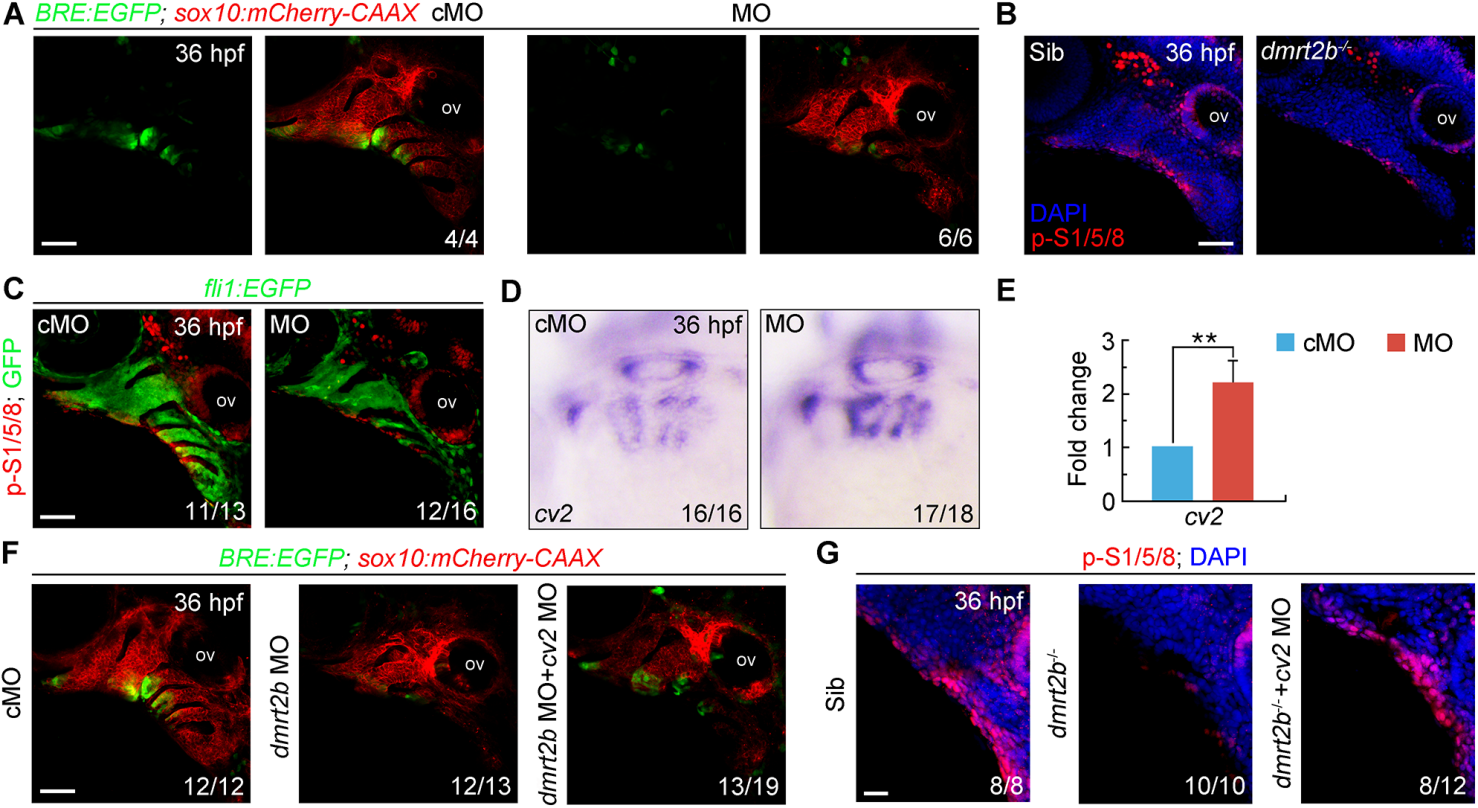
***dmrt2b* maintains BMP signaling in the pharyngeal region by inhibiting *cv2* expression.** (A) Expression of BRE-driven EGFP in the pharyngeal region of *Tg(BRE:EGFP;sox10:mCherry-CAAX)* transgenic embryos injected with 4 ng cMO or *dmrt2b* MO at 36 hpf. (B,C) p-Smad1/5/8 levels were decreased in the pharyngeal region of *dmrt2b* mutants (B) and morphants (C) at 36 hpf. Embryos were stained with the indicated antibodies. Nuclei were counterstained with DAPI (blue). (D,E) The expression of *cv2* in *dmrt2b* morphants were examined by *in situ* hybridization (D) and qRT-PCR (E). All data are presented as the mean of three independent experiments. Error bars represent s.d. Significance was analyzed using unpaired *t*-tests. **P<0.01. (F) Co-injection of 100 pg *cv2* MO with 4 ng *dmrt2b* MO partially rescued the EGFP expression in *Tg(BRE:EGFP;sox10:mCherry-CAAX)* transgenic embryos. (G) p-Smad1/5/8 levels were partially rescued in the pharyngeal region of *dmrt2b* mutants co-injected with 100 pg *cv2* MO at 36 hpf. Embryos were stained with the indicated antibodies. Nuclei were counterstained with DAPI (blue). ov, otic vesicle; p-S1/5/8, phosphorylated Smad1/5/8. Scale bars: 50 µm (A–C,F), 20 µm (G).

BMP signaling is essential for CNC cell proliferation and chondrogenic differentiation during pharyngeal cartilage development (Ning et al., 2013; Retting et al., 2009; Yoon et al., 2005). Therefore, we mated *Tg(sox10:mCherry-CAAX)* with *Tg(BRE:EGFP)*, a BMP signaling reporter transgenic line (Laux et al., 2011), to examine whether inactivation of *dmrt2b* affects BMP signaling. In the *dmrt2b* morphants, there was decreased fluorescence intensity in the pharyngeal arches, implying loss of *dmrt2b* attenuates BMP signaling in CNC cells (Fig. 5A). To further confirm this result, we examined the expression level of endogenous phosphorylated Smad1/5/8 (p-Smad1/5/8), the intracellular effectors of BMP signaling, in *dmrt2b* mutants and *Tg(fli1:EGFP)* embryos injected with 4 ng *dmrt2b* MO, and found a significantly decrease of p-Smad1/5/8 level in pharyngeal region when *dmrt2b* was depleted (Fig. 5B,C). Thus, we established that *dmrt2b* is required to maintain BMP signaling in CNC cells.

BMP genes, including *bmp2a*, *bmp2b*, *bmp4* and *bmp5*, are expressed in the endodermal pouches during pharyngeal arch development (Holzschuh et al., 2005). However, the expression of these BMP genes was not obviously decreased in *dmrt2b*-depleted embryos (data not shown). Another possibility is that *dmrt2b* promotes BMP signaling in the pharyngeal region by inhibiting the expression of some BMP antagonists. In zebrafish embryos, several BMP antagonist genes like *noggin3*, *follistatin*, *chordin* and *chordin-like 2*, have been found to be expressed in the pharyngeal region, but only *crossveinless 2* (*cv2*) is specifically expressed in the endodermal pouches during cartilage development (Ning et al., 2013; Rentzsch et al., 2006). It has been reported that Cv2 functions as a secreted BMP inhibitory protein during human chondrogenic and osteogenic differentiation (Binnerts et al., 2004). In the loss-of-function experiments, we observed that the expression of *cv2* in the pharyngeal pouches was significantly higher in *dmrt2b* morphants compared to control embryos (Fig. 5D,E). Importantly, BMP signaling in the CNC cells was partially restored by co-injecting 100 pg *cv2* MO into the *dmrt2b* morphants or mutants (Fig. 5F,G). Together, these data indicate that *dmrt2b* inhibits *cv2* expression in the pharyngeal pouches, thereby maintaining BMP signaling and facilitating CNC cell proliferation and differentiation.

## DISCUSSION

The interaction of different tissues plays vital roles in organogenesis during embryo developemt (Zorn and Wells, 2009). For example, in zebrafish, *wnt2bb* is expressed in restricted bilateral domains in the lateral plate mesoderm and directly induces the adjacent endoderm to form liver anlage (Ober et al., 2006). Recent evidence suggests that trachea-derived Decapentaplegic, the main bone morphogenetic protein ligand in *Drosophila*, is required for adult midgut homeostasis (Li et al., 2013). During craniofacial cartilage development, once the CNC cells reach their destination in the arches, signals from surrounding endodermal pouches such as FGF, BMP and CXCL12, direct the final cell fate (Crump et al., 2004; David et al., 2002; Holzschuh et al., 2005; Ning et al., 2013; Nissen et al., 2003; Boer et al., 2015; Olesnicky Killian et al., 2009). In this study, we find that *dmrt2b* is expressed in the endodermal pouches and loss of *dmrt2b* impairs pharyngeal cartilage formation. *dmrt2b* plays a critical role in the condensation of postmigratory CNC cells by promoting *cxcl12b* expression. We also provide evidence that *dmrt2b* is required for CNC cell proliferation and chondrogenic differentiation due to its ability to suppress *cv2* expression and, thus, maintain BMP signaling in pharyngeal regions. Therefore, this study demonstrates *dmrt2b*-mediated tissue–tissue interactions are essential for pharyngeal skeleton development. *dmrt2b* mutants also exhibit severe neurocranial defects and pericardial edema, indicating the possibility that *dmrt2b* is expressed at relatively low levels in other regions beyond endodermal pouches.

Members of the Dmrt family are generally associated with sex determination, but mouse *Dmrt2* is not essential for sexual differentiation (Seo et al., 2006). Mouse *Dmrt2* and its homologue gene, zebrafish *terra/dmrt2a*, have shown to be expressed specifically in developing smites and function in somitogenesis (Meng et al., 1999; Saúde et al., 2005; Seo et al., 2006). Zebrafish *dmrt2a* is also required for left–right asymmetric organ positioning (Matsui et al., 2012; Saúde et al., 2005), while this left–right function is not conserved in mouse (Lourenço et al., 2010). Interestingly, in mouse *Dmrt2* mutants, the somite patterning defects were gradually recovered during embryonic development, but the axial skeletal and rib malformations were evidently induced by the lacking of *Fgf4* and *Fgf6* expression in the myotome, suggesting a non-cell autonomous role of *Dmrt2* in controlling skeletal development (Seo et al., 2006). In our experiments, *dmrt2b* is found to be essential for pharyngeal skeleton development. Multiple lines of evidence support the idea that *dmrt2b* functions in a similar non-cell-autonomous manner by transferring developmental signals from pharyngeal endoderm to postmigratory CNC cells. (1) The expression of *dmrt2b* can be specifically detected in endodermal pouches by *in situ* hybridization experiments during pharyngeal cartilage development. (2) The endodermal pouches in *dmrt2b* depleted embryos are normally developed, indicating that the skeletal defects in the pharyngeal region are not the secondary effects of abnormalities of endodermal pouch development. (3) The experimental inhibition of *dmrt2b* function gene in pouches gives rise to obvious defects in head cartilage formation. (4) Inactivation of *dmrt2b* leads to obviously altered expression of *cxcl12b* and *cv2* in endodermal pouches, which results in disorganized arches and proliferation and differentiation defects in CNC cells. (5) Importantly, co-injection of *cxcl12b* mRNA or *cv2* MO into the *dmrt2b* morphants can partially recover the condensation defects or the decrease of BMP signaling in CNC cells. All these observations imply that *dmrt2b* regulates craniofacial skeleton development through tissue–tissue interactions. Although mouse *Dmrt2* mutant does not exhibit obvious craniofacial abnormalities (Seo et al., 2006), our findings will help to understand the developmental differences of craniofacial skeletons between lower vertebrates and mammals.

CXCL12, also known as stromal derived factor 1 (SDF-1), signals via its cognate receptor CXCR4 and plays a key role in many cellular processes including hematopoiesis, organogenesis and vascularization (Cheng et al., 2014; Teicher and Fricker, 2010). In mouse and chick embryos, *Cxcl12* is expressed in the lateral ectoderm and pharyngeal endoderm at early stages of CNC cell migration, while *Cxcr4* is expressed in migrating pharyngeal NC cells (Escot et al., 2016). Defective CXCR4 signaling impedes the migration of CNC cells into pharyngeal arches and leads to anomalies of the lower jaw and hyoid bone (Escot et al., 2016). In zebrafish embryos, *cxcl12b*, but not *cxcl12a*, is expressed within the domain of CNC cell migration from 14-17 hpf and in the endodermal pouches during pharyngeal arch morphogenesis (Olesnicky Killian et al., 2009). Unlike mouse mutants, *cxcl12b* or *cxcr4a* morphants display only a mild migration phenotype as most CNC cells arrive at the pharyngeal arches. Disruption of Cxcl12b/Cxcr4a signaling in zebrafish also results in the failure of CNC cells to fully condense within the pharyngeal arches, which is thought to be secondarily caused by the aberrant migration of CNC cells (Olesnicky Killian et al., 2009). Interestingly, loss of *dmrt2b* gives rise to decreased expression of *cxcl12b* and disorganized cells within the arches that resemble the defects observed in *cxcl12b* morphants, suggesting that *cxcl12b* is genetically downstream of *dmrt2b* during pharyngeal NC development. In addition, the expression of *dmrt2b* in the pharyngeal region is not detected until 18 hpf, and the expression of *cxcl12b* starts to decrease at 24 hpf, when the CNC cells have already migrated into pharyngeal arches. These observations would explain the lack of CNC cell migration defects in *dmrt2b* mutants, and raise the provocative idea that Cxcl12b/Cxcr4a signaling may function directly in pharyngeal NC condensation. Moreover, the disorganized arch phenotype could be rescued by injecting *cxcl12b* mRNA into *dmrt2b* morphants, suggesting that *cxcl12b* is the major downstream target of *dmrt2b* for regulating CNC cell condensation.

Cv2 displays opposing effects on BMP signaling depending on the biological context. Cv2 has been shown to potentiate BMP signaling during mouse organogenesis (Ikeya et al., 2006), and crossvein formation in the fly wing (Conley et al., 2000; O'Connor et al., 2006; Ralston and Blair, 2005), but functions as a BMP antagonist during endothelial cell differentiation (Moser et al., 2003), frog embryogenesis (Ambrosio et al., 2008; Coles et al., 2004) and human chondrogenic and osteogenic differentiation (Binnerts et al., 2004). In zebrafish, loss of *cv2* via MO-mediated knockdown results in reduced BMP signaling and dorsalized phenotypes during gastrulation (Rentzsch et al., 2006). In contrast, in our experiments, co-injection of *cv2* MO into *dmrt2b* morphants could partially recover the reduction of BMP activity in the pharyngeal arches, suggesting that Cv2 antagonizes BMP activity during lower jaw development. Indeed, the full-length zebrafish Cv2 protein acts as an inhibitor of BMP signaling and can be converted from an anti- to a pro-Bmp factor by proteolytic cleavage (Rentzsch et al., 2006). However, whether Cv2 protein can be cleaved in the pharyngeal region remains to be determined.

BMP signaling has long been recognized as an essential signal for neural crest cell specification and migration (Kanzler et al., 2000; Nie et al., 2006; Tribulo et al., 2003). During early craniofacial development, BMP signaling is required for the dorsal-ventral (DV) patterning of the pharyngeal arches (Alexander et al., 2011; Bonilla-Claudio et al., 2012; Zuniga et al., 2011). Previous studies show that, in zebrafish, the requirement of BMP activity for ventral arch development only occurs within a narrow time window from 17 to 24 hpf, a period just after CNC cell migration and before the establishment of arch primordia (Alexander et al., 2011). Not surprisingly, in *dmrt2b* mutants, no obvious defects of CNC cell specification, migration and DV patterning were found, as *dmrt2b* is expressed in the pharyngeal endoderm as early as 18 hpf and regulates *cv2* expression after 24 hpf. By contrast, our studies reveal a significant role of *dmtr2b* in CNC cell proliferation and chondrogenic differentiation by maintaining BMP activity in pharyngeal arches via suppressing *cv2* expression. This is supported by previous findings that, after arch primordia are established, inactivation of BMP signaling leads to poor proliferation and impaired differentiation of pharyngeal chondrogenic progenitors (Ning et al., 2013).

## MATERIALS AND METHODS

### Zebrafish lines

Wild-type (Tuebingen), *Tg(sox17:GFP), Tg(fli1:EGFP)*, *Tg(sox10:EGFP*), *Tg(sox10:mCherry-CAAX), Tg(BRE:EGFP)*, *Tg(nkx2.3:mCherry)* and *Tg(nkx2.3:KalTA4-p2a-mCherry)* zebrafish lines were maintained under standard laboratory conditions. Embryos were obtained from natural zebrafish matings, raised in Holtfreter's solution at 28.5°C, and staged by morphology as previously described (Kimmel et al., 1995). All zebrafish experiments were approved by and carried out in accordance with the Animal Care Committee at the Institute of Zoology, Chinese Academy of Sciences (Permission number: IOZ-13048).

### Generation of *dmrt2b* mutants

The zebrafish *dmrt2b* mutant was generated using the CRISPR/Cas9 system. The *dmrt2b* gRNA was designed using ZiFiT Targeter (http://zifit.partners.org/ZiFiT/ChoiceMenu.aspx) and the targeting sequence was 5′-GGTTGCAGACTGCGCCGGAC-3′. The Cas9 mRNA and gRNA were prepared as previously described (Wei et al., 2017) and co-injected into one-cell stage wild-type embryos. For genotyping analysis, the genomic DNA was isolated and used as template for amplification of gRNA targeted sequences with the forward primer 5′-CAATCACTGCTGCATTCCGAC-3′ and the reverse primer 5′-TGTCTCCGTAGGGCGACTTGA-3′. Then the amplified fragments were identified with Sanger DNA sequencing.

### Constructs

Total RNA was extracted from wild-type embryos at 36 hpf using TRIzol reagent (15596018, Invitrogen) and reverse transcribed using the Rever Tra kit (Toyobo). The resulting total cDNAs were used to amplify required segments of *dmrt2b* (NM_001079976.1), *nkx2.3* (NM_131423.1), and *cv2* (NM_001020487.2) transcripts by using the primers listed below and then cloned into the EZ-T^TM^ vector (T168-101, GenStar).

Primers: *dmrt2b*, forward primer 5′-CGCTGTCAGACCCAATCATG-3′ and reverse primer 5′-CTTTACTAGCACCCTCC-3′; *nkx2.3*, forward primer 5′-GATTTCAGGCACCATCGTGG-3′ and reverse primer 5′-GCTGGGTTGCACTGGCACTA-3′; and *cv2*, forward primer 5′- AGGCAAAGACAACCGGACATCTA-3′ and reverse primer 5′- AAAGTCATTCTGTAATCCCAGTC-3′.

For rescue studies, the full length cDNAs of *dmrt2b* and *cxcl12b* were amplified by RT-PCR using the following primers and then cloned into the pCS2-Flag vector.

Primers: *dmrt2b*, forward primer 5′-CCGGAATTCATGTCCACTAAAGCGGATAG-3′ and reverse primer 5′-CGCGGATCCTTATCTCATGAGCAGTGCCT-3′; *cxcl12b*, forward primer 5′-GCCACCATGGATAGCAAAGTAGTAG-3′ and reverse primer 5′-TATCTCGAGCTCTGAGCGTTTCTTC-3′.

### RNA synthesis, MOs, microinjections and whole-mount *in situ* hybridization

Digoxigenin-UTP-labeled RNA probes were synthesized *in vitro* from linearized plasmids using the MEGAscript® Kit (Ambion) according to the manufacturer's instructions. *In vitro* synthesis of *dmrt2b* mRNA was performed from linearized plasmids using the mMESSAGE mMACHINE Kit (Ambion). The standard control morpholino (cMO) (5′-CCTCTTACCTCAGTTACAATTTATA-3′), *sox32* MO (5′-CAGGGAGCATCCGGTCGAGATACAT-3′) (Dickmeis et al., 2001) and *cv2* MO (5′-TTACTGGAGGAGACAGACACAGCAT-3′) (Rentzsch et al., 2006) were used as previously described. The *dmrt2b* MO (5′-CTTTCTTACCCTGTTGATGAGAACA-3′) was designed and synthesized by Gene Tools. Microinjections and whole-mount *in situ* hybridization were performed as previously described (Jia et al., 2009).

### RNAscope assay combined with immunofluorescence staining

RNAscope assay was conducted by using the RNAscope Flurescent Multiplex Reagent Kit [320850, Advanced Cell Diagnostics (ACD)]. Embryos were treated with Pretreat 3 buffer for 10 min at room temperature before hybridization. For the hybridization, *dmrt2b* RNAscope probe (510211-C3, ACD) and Amp4 Alt A-FL (320855, ACD) were used. Immediately following this, immunofluorescence staining was performed to detect proteins with GFP antibody (1/1000; A-11122, Invitrogen) and DAPI (1/3000; 10236276001, Sigma-Aldrich) as previously reported (Gross-Thebing et al., 2014; Wang et al., 2012). Finally, the embryos were washed in PBST and images were taken using a Nikon A1R+ confocal microscope.

### Whole-mount immunofluorescent staining and TUNEL assays

Whole-mount immunofluorescent staining was performed as previously reported (Ning et al., 2013). Briefly, embryos were fixed with 4% phosphate-buffered paraformaldehyde and washed with 0.3% Triton X-100 and 0.1% Tween-20 in PBS for 20 min before immunostaining. The embryos were stained with the indicated antibodies, including anti-GFP (1/1000; A-11122, Invitrogen), anti-GFP (1/1000; A-11120, Invitrogen), anti-Collagen type II (Col2) (1/100; II-116B3, Developmental Studies Hybridoma Bank), anti-pH3 (1/1000; 3377, Cell Signaling Technology) and anti-p-Smad1/5/8 (1/200; 9511, Cell Signaling Technology). All immunofluorescent images were captured using a Nikon A1R+ confocal microscope with the same settings for all experiments.

TUNEL assays were performed using the In Situ Cell Death Detection Kit, TMR red (12156792910, Roche) according to the manufacturer's instructions. DAPI was used to visualize nuclei.

### Alcian Blue staining

Embryos were fixed in 4% paraformaldehyde overnight at 4°C. Immediately following, fixed embryos were washed in distilled water with 0.1% Tween-20 for 8 h. The embryos were then stained with Alcian Blue staining buffer (0.015% Alcian Blue, 80% ethanol, and 20% acetic acid) overnight at room temperature and then de-stained in 70% ethanol/30% acetic acid. Next, the embryos were rehydrated through a graded series of alcohols to distilled water and then treated with 0.5% trypsin (0458, AMRESCO) in supersaturated borax at room temperature until the tissues were soft enough to dissect. The embryos were then transferred to 1% KOH for 30 min and then washed in distilled water with 0.1% Tween-20 twice for 5 min each. Finally, the embryos were dehydrated with a graded series of glycerol solutions and dissected for imaging.

### Time-lapse imaging

Embryos were anaesthetized and embedded in 0.8% low-melt agarose (0815, AMRESCO) at the indicated time points for live imaging with a Nikon A1R+ confocal microscope (20× dry, 40× dry or 60× oil objectives). All confocal stack pictures were processed using Nikon NIS-Elements AR 4.13.00 software.

### Semi-quantitative and quantitative RT-PCR

Semi-quantitative RT-PCR and quantitative RT-PCR were performed as previously described (Wei et al., 2017). For semi-quantitative RT-PCR analysis, *dmrt2b* MO interfered products were amplified using forward primer 5′-AGCCTTTGTTAGACAGATA-3′ and reverse primer 5′-ACGGGAAGAAATACGG-3′. To detect endogenous *dmrt2b* mRNA, forward primer 5′-AGTCGCCTTCTAGGAAACATC-3′ and reverse primer 5′-CAGTATTGGAGGAATGTCTTG-3′ were used. For quantitative analysis, *cxcl12b* and *cv2* were amplified using SYBR® Premix Ex Taq^TM^ dye (Takara) in Analytic Jena PCR qTOWER 2.2 system using the following primers: 5′- CTCCACCCTCAACACCG-3′ and 5′-TTTAGATACTGCTGAAGCCATT-3′ for *cxcl12b*; 5′-CCAAACGCCACAATCAAC-3′ and 5′-CACTTCTCCTGCTTACACTCC-3′ for *cv2*. In both experiments, *β-actin* were amplified as internal controls using primers as previously described (Wei et al., 2017).

### Statistical analysis

The cell shape of CNC cells was indicated by length/width ratio and the front eight rows of CNC cells in the first and second pharyngeal arches were analyzed. ImageJ software was used to measure the distance. All experiments were performed in triplicate and unpaired *t*-test was employed to analyze all data sets. Results were considered statistically significant at *P*<0.05.

## Supplementary Material

Supplementary information
