# Supplementary material for: Endodermal pouch-expressed dmrt2b is important for pharyngeal cartilage formation
Source: Biol Open. 2018 Oct 19;7(12):bio035444. doi: 10.1242/bio.035444 (PMC6310889; doi:10.1242/bio.035444)
Supplement: Supplementary information [file biolopen-7-035444-s1.pdf]

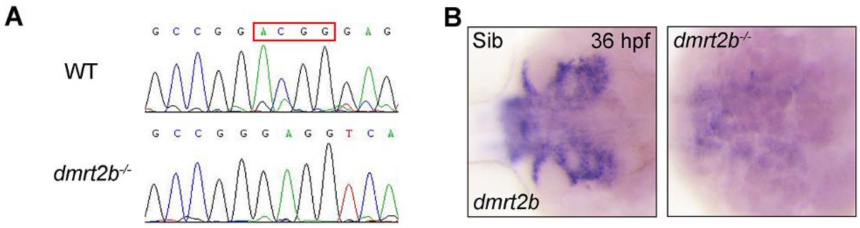

Figure S1. Validation of *dmrt2b* mutant. (A) The sequence of *dmrt2b* in wild-type and *dmrt2b* mutant. (B) Detection of *dmrt2b* expression using *in situ* hybridizations in siblings and *dmrt2b* homozygous mutants.

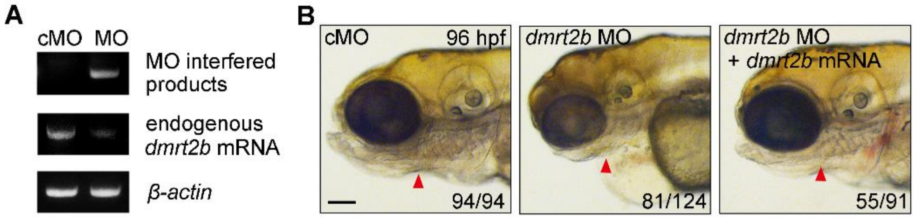

Figure S2. *dmrt2b* is required for craniofacial skeleton formation. (A) The efficiency of *dmrt2b* MO. Wild-type embryos were injected with 4 ng standard cMO or *dmrt2b* MO at the one-cell stage and harvested at 24 hpf for semi-quantitative RT-PCR analysis. (B) Morphology of head skeleton in embryos injected with cMO, *dmrt2b* MO or both *dmrt2b* MO and *dmrt2b* mRNA. Red arrowheads indicate branchial arches. Scale bar, 100 μm.

Biology Open • Supplementary information

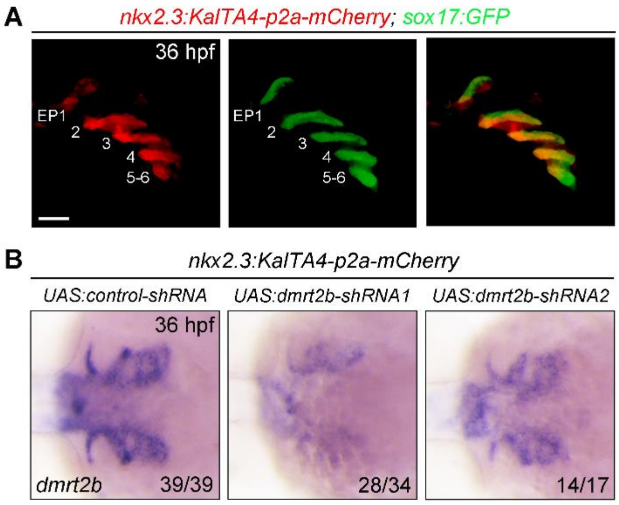

Figure S3. Validation of *dmrt2b* shRNAs using KalTA4-UAS system. (A) Live confocal images of *Tg(nkx2.3:KalTA4-p2a-mCherry;sox17:GFP)* double transgenic embryos at 36 hpf. The endodermal pouches are labeled in the left two panels. EP, endodermal pouch. Scale bar, 50 μm. (B) The knockdown efficiency of *dmrt2b*-shRNAs. *Tg(nkx2.3:KalTA4-p2a-mCherry)* embryos were injected with 50 pg indicated *UAS:shRNA-GFP* plasmids and 100 pg Tol2 transposase mRNA at one-cell stage, and then embryos with robust GFP fluorescence in the pouches were selected at 36 hpf for *in situ* hybridization.

Biology Open • Supplementary information

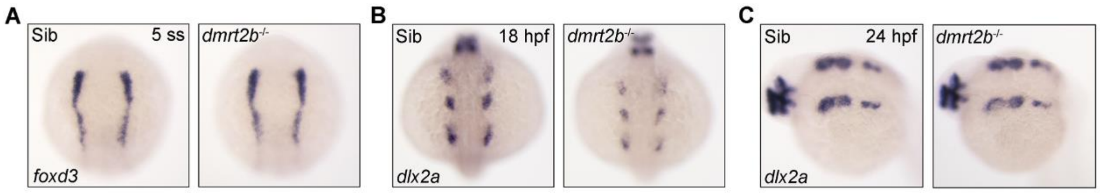

Figure S4. Loss of *dmrt2b* causes no effects on CNC specification and migration. (A) The expression of CNC specification marker *foxd3* in siblings and *dmrt2b* mutants. (B, C) The expression pattern of CNC migration marker *dlx2a* in siblings and *dmrt2b* mutants at 18 hpf (B) and 24 hpf (C). Panel A and B, dorsal views with anterior to the top. Panel C, lateral views with anterior to the left.

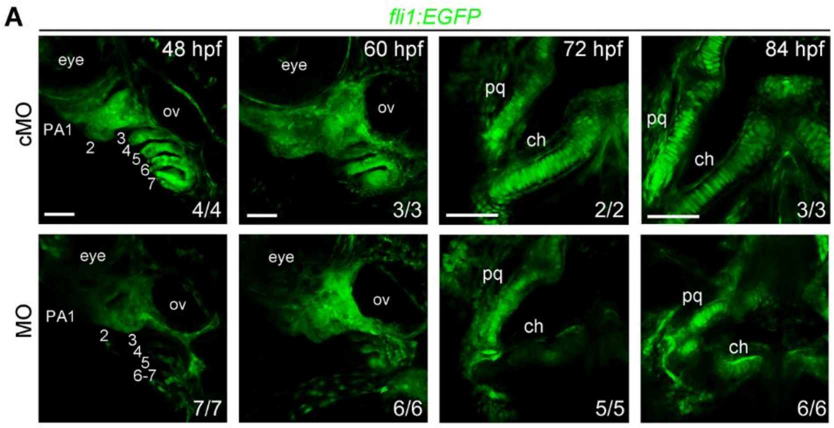

Figure S5. Time-lapse imaging of the pharyngeal region in *Tg(fil1:EGFP)* transgenic embryos. (A) Time-lapse imaging of CNC cells in *Tg(fli1:EGFP)* transgenic embryos from 48 to 84 hpf. PA, pharyngeal arch; ov, otic vesicle; pq, palatoquadrate; ch, ceratohyal. Scale bars, 50 μm for the 48 and 60 hpf and 20 μm for the 72 and 84 hpf.

Biology Open • Supplementary information

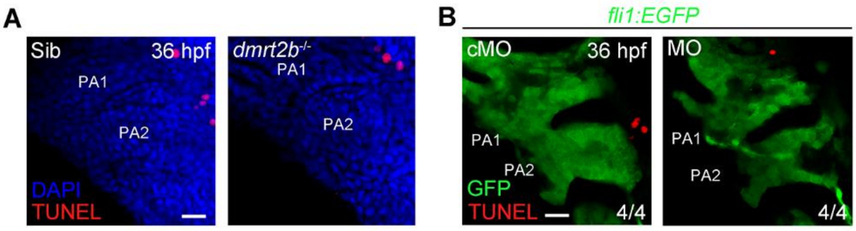

Figure S6. Inactivation of *dmrt2b* does not trigger apoptosis in pharyngeal CNC cells. (A) Detection of apoptotic cells (red) in *dmrt2b* mutants with TUNEL assay at 36 hpf. DAPI (blue) marked the nuclei. The first two pharyngeal arches (PA1 and PA2) are shown in the lateral views with anterior to the top. Scale bar, 20 μm. (B) Detection of apoptotic cells (red) in *dmrt2b* morphants with TUNEL assay in *Tg(fli1:EGFP)* transgenic embryos at 36 hpf. Scale bar, 20 μm.

Biology Open • Supplementary information
